# Early pregnancy in the horse revisited – does exception prove the rule?

**DOI:** 10.1186/s40104-015-0048-6

**Published:** 2015-12-02

**Authors:** Christine Aurich, Sven Budik

**Affiliations:** Artificial Insemination and Embryo Transfer, Vetmeduni Vienna, Veterinärplatz 1, 1210 Vienna, Austria

**Keywords:** Conceptus, Horse, Maternal recognition, Pregnancy

## Abstract

Early equine pregnancy shares many features with that of more intensively assessed domestic animals species, but there are also characteristic differences. Some of those are poorly understood. Descent of the equine conceptus into the uterine lumen occurs at day 5 to 6 after ovulation but is only possible when the embryo secretes prostaglandin E2. Although maintenance of equine pregnancy probably involves secretion of a conceptus derived anti-luteolytic factor, this agent has not been identified. Rapid growth, conceptus mobility and presence of an acellular capsule at the time of maternal recognition of pregnancy, i.e. between days 12 and 14, are prerequisites to avoid pregnancy loss. Progesterone together with 5α-pregnanes is secreted by the corpus luteum and induces the production of endometrial histotroph which is responsible for conceptus nutrition until placention. A stable contact between the outer trophoblast layer of the allantochorion and the luminal epithelium of the endometrium is not established before days 40 to 42 of pregnancy.

## Introduction

In mammals, maintenance of pregnancy depends on the continuous production of progesterone. The rule is that extension of corpus luteum lifespan beyond the length of one physiological estrous cycle requires either a luteotrophic (e.g. as in humans) or an anti-luteolytic (e.g. as in ruminants or pigs) factor which is produced by the conceptus. Although presumed by many authors [1–5] that maintenance of pregnancy in the horse will involve secretion of an anti-luteolytic factor by the conceptus, this agent has so far not been identified. Knowledge on early pregnancy in the horse thus lacks an important component. In other species the anti-luteolytic factor does not only inhibit luteolysis but is also involved in modulation of endometrial functions in preparation to pregnancy [6]. In contrast to other domestic animals, some horse-specific limitations challenge the research on early pregnancy: access to experimental animals or genital organs is mostly limited in a species where meat consumption is not common in many countries and thus slaughterhouse material is almost not readily available. Superovulatory treatment with the aim to produce multiple conceptuses is difficult in the horse, i.e. mares cannot be superovulated to a meaningful extent [7]. At present no efficient superovulatory drug for the horse is available. Furthermore, late entry of the conceptus into the uterus and limited success of in vitro produced embryos makes research on early stages of pregnancy difficult in this species. The knowledge on early equine pregnancy in some aspects is therefore quite rudimentary in comparison to other domestic animal species. Nevertheless, many mechanisms and features – some of them quite unique among domestic animals - have been well characterized. Ongoing research may eventually lead to the solution to the riddle of maternal recognition of pregnancy in the horse. In this review, the present knowledge is critically summarized.

### Current knowledge on maternal recognition of pregnancy in the horse

The horse is a seasonal breeding species with reproductive activity being associated with long days, i.e. occurring in spring and early summer. During the breeding season, cycle length is about 22 days with 5 to 7 days of oestrus. Functional luteolysis occurs on day 15 after ovulation [8]. Initiation of the luteolytic cascade in the horse was for a long time suggested to happen on day 10 after ovulation or even earlier [9, 10]. More recent research demonstrated successful transfer of day 10 embryos to mares that were either on day 10 or day 12 after ovulation. This proves that the luteolytic cascade in the non-pregnant mare is not initiated before day 12 after ovulation [11]. It can thus be concluded that the anti-luteolytic mechanism of the equine conceptus has to be active between days 12 and 14 after ovulation.

In the non-pregnant mare, luteolysis is initiated by endometrial secretion of prostaglandin (PGF)_2α_. On day 15 of the oestrous cycle, expression of cyclooxygenase 2 (COX2) by uterine epithelial cells of non-pregnant mares is markedly increased while it is inhibited in pregnant mares. Regulation of endometrial expression of COX2 is therefore considered a key event in either induction of luteolysis or maternal recognition of pregnancy in the horse [12, 13]. In agreement with the situation in other species, endometrial PGF_2α_ release is stimulated by oxytocin [13]. In the mare, there is no significant synthesis of luteal oxytocin, but oxytocin has been localized in the endometrium [14]. However, administration of exogenous oxytocin was unable to induce endometrial PGF_2α_ release during early pregnancy despite increased expression of endometrial oxytocin receptors. Therefore, a paracrine-autocrine system involving endometrial oxytocin and PGF_2α_ most likely accelerates luteolysis in the non-pregnant mare [8].

For the horse conceptus, the signal or mechanism that inhibits luteolysis has not been identified. Unlike the ruminant conceptus, the equine conceptus does not produce interferons that inhibit endometrial PGF_2α_ release [15]. The fact that equine conceptuses produce estrogens in high amounts from day 10 of pregnancy onwards [16] has stimulated research on estrogens as a potential anti-luteolytic agent in this species. This hypothesis could not be supported because estrogens – when provided at physiological concentrations - did not extend corpus luteum lifespan in horse mares [1, 4, 5]. The nature and origin of the antiluteolytic signal in the horse conceptus thus differs from domestic ruminants and pigs. In 1989, Sharp et al. [2] published evidence that the antiluteolytic agent secreted by the equine conceptus has a molecular weight between 1,000 and 6,000. However, molecules fitting into this molecular mass like PGE_2_ or insulin failed to prolong lifespan of the corpus luteum in cyclic mares when infused into the uterine lumen [5, 17]. Development of an endometrial explant in vitro culture system appeared promising to support further research for identification and characterization of the equine conceptus factor responsible for maternal recognition of pregnancy [13]. Unfortunately further relevant results have not been published.

Before and on day 14 of pregnancy, the equine yolk sac produces a characteristic pattern of proteins that completely changes thereafter. It was suggested that one or more of these proteins might be involved in the anti-luteolytic mechanism of the horse conceptus [3], but this has never been proven. The switch in protein expression by the yolk sac around day 14 is most likely associated with development of the mesoderm with its blood-forming islets [3, 18]. Uterocalin which has mainly received consideration as an endometrial protein (see below) is also expressed in conceptus tissue with decreasing expression between days 8 and 14 of pregnancy [19].

Persistence of the corpus luteum is also seen in a certain percentage of non-pregnant mares after introduction of a glass marble [20] or fluid-filled rubber ball [21] into the uterine lumen during the first days after ovulation. The presence of a spherical intrauterine device has thus been suggested to resemble the presence of a conceptus by exerting contact or pressure directly on the uterine wall [21]. This may induce changes in the endometrial epithelia similar to those induced by presence of a conceptus. Interestingly, the effect seems to depend on adequate perfusion and drainage of the endometrium and is less effective in aged mares [22]. These results suggest that the embryonic signal for maternal recognition of pregnancy in the horse might be at least in part mechanical rather than secretory in origin. This assumption was further supported by modulation of prostaglandin production and prolonged corpus luteum lifespan reported after intrauterine administration of different plant oils into the uterine lumen of luteal phase mares [23]. The authors could not exclude the possibility that physical interference with the endometrium was involved in this phenomenon. However, in contradiction to this hypothesis, intrauterine administration of mineral oil did not prevent luteolysis.

### Sources of progestin during equine pregnancy

In domestic animal species, pregnancy is maintained by secretion of progesterone from the corpus luteum, the placenta or a combination of both. The situation is more complicated in pregnant mares where not only different sources for progestin secretion exist, but also a variety of progestins as well as estrogens are secreted [24, 25]. From ovulation until approximately day 40 of pregnancy, progestins and estrogens are solely secreted from the primary corpus luteum [26–29]. Besides progesterone the progestins 5α-pregnane-3,20-dione and 3β-hydroxy-5α-pregnan-20-one are detectable in the circulation [30]. Progestin concentrations in blood of mares increase rapidly after ovulation and peak around day 5 of pregnancy. From then onwards concentrations in maternal plasma gradually decline suggesting only a weak luteotrophic signal in early pregnant mares [31]. A second increase in concentration of progestin in maternal plasma around day 40 of pregnancy is based on the formation of secondary corpora lutea. Their formation is initiated by secretion of equine chorionic gonadotropin (eCG) from the endometrial cups from day 37 after ovulation [32]. A further support of pregnancy arises with the start of placental steroid synthesis around day 60 of gestation. Placental steroids again consist of different progestins, mainly 5α-pregnanes. From this time, circulating concentrations of progestin in the pregnant mare are considered a mixture of luteal and placental progestins until the feto-placental unit becomes the only source of progestins from day 160 of gestation onwards [30], when the function of the primary corpus luteum and secondary corpora lutea ceases [33].

### Development of the early equine conceptus

**Fig. 1:**
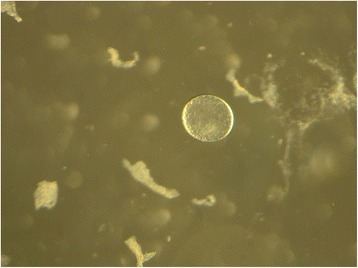
Blastocyst collected from the uterus of a mare on day 7 after ovulation. The zona pellucida is clearly visible

In the horse, fertilization rate following natural service is greater than 90 % [34]. First cleavage of the fertilized equine oocyte occurs approximately 24 h after fertilization, subsequent divisions of the blastomeres follow at 12- to 24-h intervals [35]. Morphological reorganization of the nucleolus coinciding with activation of embryonic transcription takes place at the 6- to 8-cell stage, i.e. at the fourth embryonic cell cycle [36]. The early equine zygote is characterized by marked asymmetry in the distribution of cellular organelles and inclusions. This is suggested to contribute to the more ellipsoidal shape of the early equine embryo [37]. At the 8- to 16-cell stage, tight junctions between individual blastomeres are formed, causing aggregation and subsequent compaction of the cells. Thereafter, individual blastomeres cannot longer be identified, continuous cell division and tight junction formation leads to formation of a compact morula which consists of at least 32 blastomeres [38]. In the horse, the compact morula is the latest developmental stage found in the oviduct [39, 40]. It will develop into a blastocyst (Fig. 1) after entering the uterine lumen at approximately 6 days after ovulation. In the horse, transport of the embryo from the oviduct into the uterine lumen is selective and depends on the release of prostaglandin E_2_ by the conceptus shortly before the time of entering the uterus, i.e. on days 5 and 6 after ovulation [41, 42]. While segregation of the inner cell mass from the trophoblast at the time of blastocyst formation in ruminant and pig conceptuses is rapid and distinct, the cells of the inner cell mass in horse blastocysts remain much more dispersed. Differentiation between morulae and early blastocysts may therefore be difficult [37]. Already at the time of blastocyst formation, conceptus size is highly variable [43, 44]. It is influenced not only by day of pregnancy, but also by factors such as age of mare, the method of processing the semen used for breeding and number of ovulations per estrus [44–46]. Despite the fact that horses are seasonal breeders, conception rate as well as conceptus quality and growth is not impaired in mares that are spontaneously cyclic during the non-breeding season [46].

In contrast to ruminants and pigs, the increase in size of the equine conceptus is initially caused mainly by water influx and only to a minor degree by multiplication of cells [38]. During blastocyst expansion the formation of an osmotic gradient by α1/β1 Na+/K + −ATPase is the driving force of water influx into the blastocoel of equine embryos [47, 48]. After completion of endoderm formation around day 8, the blastocoel is termed yolk sac. Starting around day 10, osmolarity of the yolk sac fluid decreases. The yolk sac fluid is markedly hypotonic until about day 18 when osmolarity gradually increases [37]. Hypoosmolarity within the yolk sac seems to contradict the hypothesis of a Na+/K+ trans-trophoblast gradient responsible for blastocoel expansion before day 8 [49]. The control of equine yolk sac expansion is most probably mediated by changes in the permeability of the apical ectodermal membrane to water through differences in the abundance of aquaporin (AQP) 5. Vasopressin in the yolk sac could participate in the regulation of AQP5 function in a similar manner as in kidney collecting ducts [50, 51]. Subtrophoblastic compartments described in equine blastocysts seem to undergo a sharp increase in tonicity relative to the interior of the yolk sac, forming a third compartment which might be responsible for maintenance of the ion gradient in the equine conceptus larger than 6 mm in diameter [52].

**Fig. 2:**
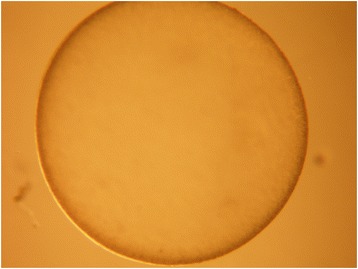
Horse conceptus collected from the uterus of a mare on day 7 after ovulation with the acellular capsule being clearly visible

The horse conceptus remains spherical much longer than the ruminant or pig conceptus which loses the spherical shape soon after hatching from the zona pellucida. From day 6 until approximately day 23 of pregnancy, the horse conceptus is surrounded by an acellular mucin-like glycoprotein capsule (Fig. 2) [38, 53–55]. Expansion of the capsule facilitates shedding of the zona pellucida. The capsule continues the protective function of the zona pellucida and is thus considered essential to the continuation of pregnancy [56]. Transfer of embryos into synchronous recipient mares after removal of the capsule dramatically impairs pregnancy rates [55]. The progestin-dependent endometrial protein uterocalin functionally correlates with capsule formation and persistence [57] which is in agreement with the finding that in vitro produced horse embryos fail to form a normal acellular capsule [58]. Nevertheless, addition of uterocalin to the culture media did not result in physiologic formation of a capsule in in vitro produced equine embryos [59]. Therefore, contact with the complex uterine environment seems to be essential for capsule formation.

**Fig. 3:**
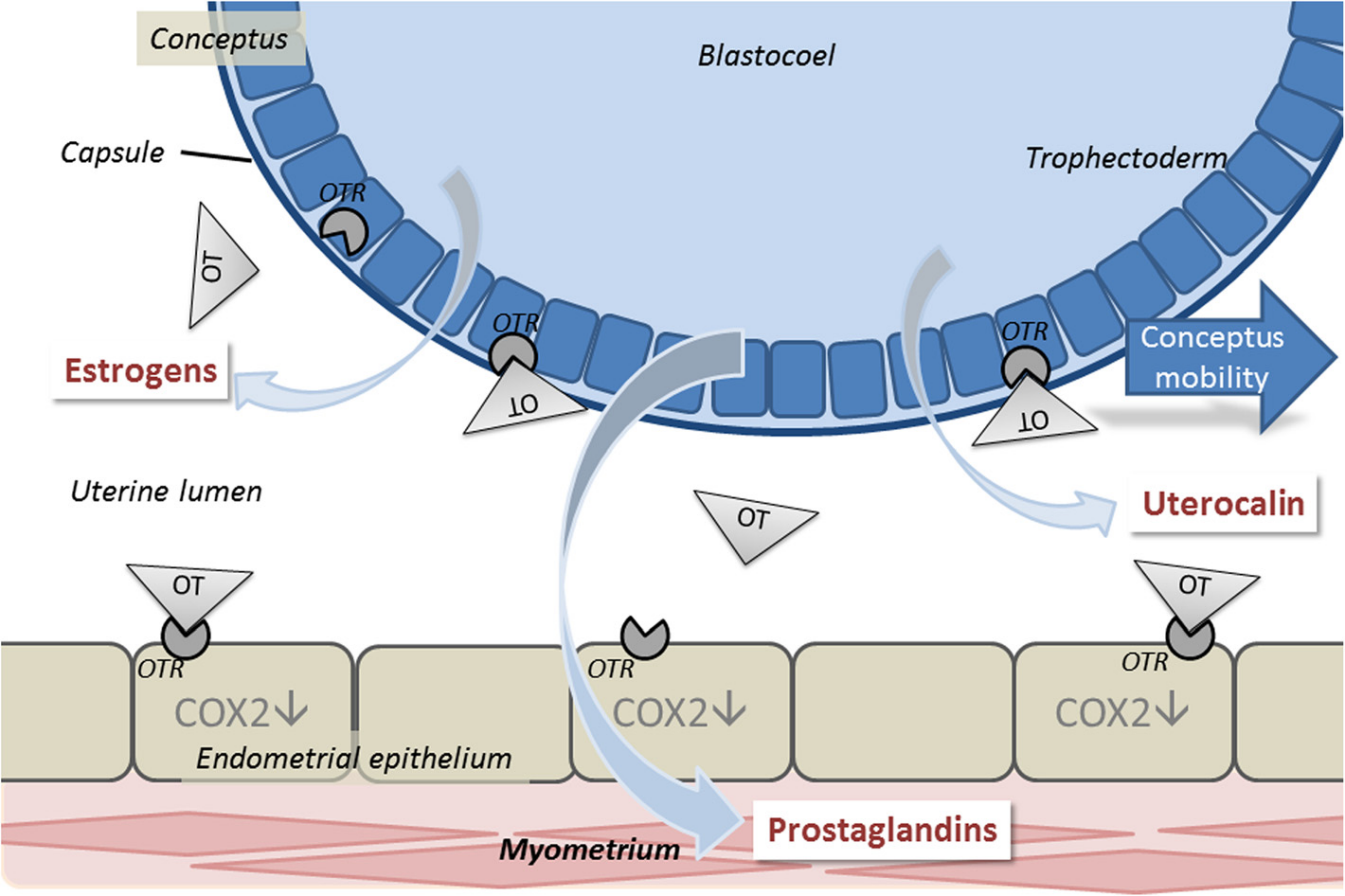
Schematic representation of interactions between the conceptus and the uterus as currently proposed at the time of maternal recognition of pregnancy on days 12/13 after ovulation: The conceptus propels through the uterine lumen dependent on the action of conceptus-derived prostaglandins on the myometrium. In addition, the conceptus secretes estrogens and arginine into the uterine lumen. Endometrial oxytocin (OT) stimulates conceptus growth by action on OT receptors (OTR) in the trophectoderm. Due to down-regulation of cyclooxygenase 2 (COX2) in the endometrial epithelium, endometrial oxytocin cannot stimulate endometrial synthesis of prostaglandin F_2α_, therefore corpus luteum function is maintained

**Fig. 4:**
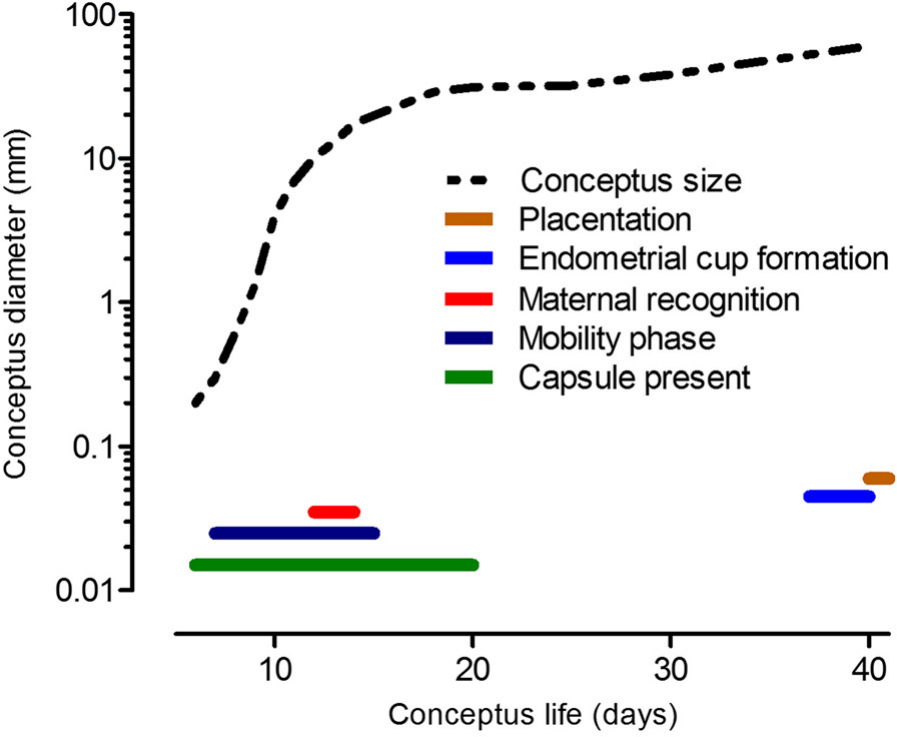
Mean conceptus diameter (mm) of the horse conceptus between days 7 and 40 after ovulation and time of some significant events that are involved in the establishment of equine pregnancy

Between days 10 and 15, i.e. at the time of maternal recognition of pregnancy, the equine embryo moves constantly through the uterine cavity (Figs. 3 and 4). This feature is suggested to compensate for the relatively small trophoblast surface area in this species [60, 61]. Restriction of conceptus mobility to only part of the uterine lumen results in failure of pregnancy in the horse [62]. Embryonic mobility depends on local peristaltic contractions of the myometrium that are most likely induced by prostaglandins synthesized and secreted from the conceptus itself [63–65]. Besides mobility, adequate size of the conceptus is a prerequisite for maternal recognition of pregnancy [60, 61] while retarded growth and inappropriate development are considered major reasons for early pregnancy loss in mares [66, 67, 68, 69, 76]. However, it has to be considered that active migration of the spherical blastocyst also occurs in ruminant and pig embryos after hatching and before development into tubular and then filamentous forms [70] and is therefore not completely unique to the equine species.

In horses, the yolk sac is suggested to be an important source of nutrition for the conceptus during the first 3 to 4 weeks of pregnancy [71]. It thus persists beyond developmental stages when it becomes nonfunctional in conceptuses of most domestic animals. As a morphological structure, the yolk sac is often recognized at parturition of the foal. Architecture of the conceptus at the time of fixation, i.e. around day 16 of pregnancy, is believed to play an important role in its orientation within the uterine lumen [71]. Blister-like structures formed between ectoderm and mesoderm in the trilaminar part of day 14 and day 16 conceptuses may be involved [18].

### Endometrial function in the mare during early conceptus development

In all mammals, establishment and maintenance of pregnancy depends on the presence of progesterone. In the mare, the presence of progesterone is a prerequisite for conceptus mobility, fixation at the basis of one uterine horn and orientation within in the uterus [72]. Expression of progesterone receptors in the trophoblast may allow for direct effects of progesterone on the conceptus [73, 74]. However, the main task of progesterone is the preparation of the endometrium for pregnancy. Paradoxically, this requires down-regulation of progesterone receptors in endometrial epithelia as a prerequisite for the expression of pregnancy-associated proteins [75]. Mares have a similar pattern of endometrial progesterone receptors during early pregnancy as other mammals. Progesterone receptors are absent in the endometrial epithelia from day 20 of pregnancy, but remain abundant in stromal cells [76]. Treatment of mares with a synthetic progestin from day 5 after ovulation resulted in enhanced downregulation of epithelial endometrial progesterone receptors already on day 11 after ovulation [74]. In cows, a positive relationship between concentrations of progesterone in maternal plasma and development of the embryo has been demonstrated. High concentrations of progesterone in the early postovulatory phases of the estrous cycle stimulate a stronger antiluteolytic signal [77, 78].

In many domestic animals, rodents and primates, the trophectoderm of the conceptus produces interferons (IFN) during the peri-implantation period. IFNτ (IFNT) is unique to ruminants and has been identified as their conceptus’ signal for maternal recognition of pregnancy. Besides, IFNs are involved in the regulation of uterine receptivity, decidualization, as well as placental growth and development. They induce the expression of IFN-stimulated genes in the uterus in a temporal and cell-specific manner [70]. IFNδ (IFND) has been demonstrated not only in pigs [79], but also in horses [80]. In this species, two IFND genes have been identified and are expressed between days 16 and 22 of pregnancy. This suggests involvement of IFND in conceptus-maternal interactions in the horse, but the expression occurs beyond the time of maternal recognition of pregnancy.

Duration of the pre-implantation period varies considerably among species, but is prolonged in the horse. The outer trophoblast layer of the allantochorion finally establishes a stable, microvillous contact with the luminal epithelium of the endometrium around days 40 to 42 and placentation commences thereafter [81]. Before placentation, the equine conceptus is completely dependent on nutritional support by histotroph secreted from the luminal epithelium and the endometrial glands [82]. Histotroph is produced in all mammalian uteri and consists of a complex mixture of proteins and molecules. Its production depends on progesterone action and – in sheep has been demonstrated to be IFNT-stimulated [6, 70]. At the blastocyst stage, the energy substrate for mammalian conceptuses switches from pyruvate to glucose. In sheep, concentrations of glucose and the amino acids arginine, leucine and glutamine increase in the uterine lumen between days 10 and 15 of pregnancy. This is paralleled by increased expression of specific transporters of those nutrients in the uterine epithelia. These changes are indispensable for the survival and development of the conceptus [6]. The same level of knowledge does not exist for the horse so far. However, changes at the mRNA level of the maternal endometrium during equine early pregnancy have been investigated utilizing microarray techniques. Pronounced changes occurred around the time of recognition of pregnancy. A high proportion of genes with altered transcription is regulated by estrogens, progesterone or PGE_2_. It is thus feasible that in the mare changes in mRNA abundance are also directly related to maternal progesterone secretion and/or conceptus-derived factors such as estrogens or PGE_2_. Because several of the affected genes are also involved in regulation of early gestation in species other than the horse it is suggested that a subset of genes crucial to endometrial receptivity is highly conserved among species [83, 84]. The importance of progesterone for histotroph production and maintenance of pregnancy in the horse has been long emphasized (reviewed by Sharp 2000). Similar to ruminants, a pronounced rise in progestins during the early post-ovulatory phase in pregnant mares contributes to improved development of the conceptus [45, 85] while deprivation of progesterone due to luteolysis leads to immediate changes in endometrial protein secretion [86]. Among others retinol binding protein [87], uteroferrin [88], uterocalin [82] and SLC36A2 (solute carrier family 36 (proton/amino acid symporter), member 2) [83] have been suggested to be of significance for maintenance of early pregnancy in the horse. Uterocalin has received specific interest because it has been suggested to facilitate lipid transport across the acellular embryonic capsule [82]. Histological evaluation of conceptuses collected on days 14 and 16 of pregnancy supports the hypothesis of a highly absorptive trophoblast during this time of development [18]. Furthermore, changes in the expression of a total of 42 members of the solute carrier group of membrane transport proteins were determined, 30 of them being upregulated and 12 being down-regulated. This suggests that these transporters contribute to nutrient exchange between the histotroph and the developing conceptus with unique subsets which are characteristic for different stages of conceptus development [19].

## Conclusion

The majority of information available with respect to early equine pregnancy and conceptus development supports the idea of an anti-luteolytic mechanism responsible for maintenance of corpus luteum function beyond the physiological events of the estrous cycle. Despite intensive research, the nature of the embryonic signal for luteostasis in mares remains a mystery. It may be suggested that in the horse, luteolysis is prevented by a more complex conceptus-related mechanism and not only by a single substance. The reason why such a mechanism remains undetected until now is unclear. However, it appears feasible that the rapid development of molecular biological methods will eventually allow scientists to solve the riddle.

## Abbreviations

AQP: Aquaporin
cAMP: Cycloadenosinmonophosphat
COX2: Cyclooxygenase 2
eCG: Equine chorionic gonadotropin
IFN: Interferon
mRNA: Messenger ribonucleic acid
PG: Prostaglandin
